# High-fat diet combined with dextran sulfate sodium failed to induce a more serious NASH phenotype than high-fat diet alone

**DOI:** 10.3389/fphar.2022.1022172

**Published:** 2022-09-27

**Authors:** Yan Zhou, Ya Feng, Lili Yang, Peiyong Zheng, Lu Hang, Fengru Jiang, Jianye Yuan, Lixin Zhu

**Affiliations:** ^1^ Institute of Digestive Diseases, Longhua Hospital, Shanghai University of Traditional Chinese Medicine, Shanghai, China; ^2^ Department of Colorectal Surgery, Guangdong Institute of Gastroenterology, The Sixth Affiliated Hospital, Sun Yat-sen University, Guangzhou, China

**Keywords:** non-alcoholic steatohepatitis, animal models, high-fat diet, dextran sulfate sodium, liver fibrosis

## Abstract

**Background and Aims:** Animal models are essential tools to investigate the pathogenesis of diseases. Disruption in the intestinal epithelial barrier and gut vascular barrier is an early event in the development of non-alcoholic fatty liver disease (NAFLD). Intestinal epithelial barrier can be destroyed by dextran sulfate sodium (DSS) oral administration. High fat diet (HFD)-induced non-alcoholic steatohepatitis (NASH) rat model has been widely used. Recently, the combination of HFD with DSS induced NASH model has also been reported. The present study aimed to evaluate whether this composite NASH animal model is more ideal than that induced by HFD alone.

**Methods:** Rats were divided into control, HFD and HFD combined with DSS (DSS + HFD) groups. They were fed with routine diet, high-fat diet, and HFD combined with DSS drinking, respectively, for 22 weeks. Histopathological analysis (HE staining, Oil-Red O staining, Masson staining), lipid parameters testing (TG, TC, GLU, NEFA, TRIG, LDL, HDL), testing on indicators of inflammation (TNF-α, ALT, AST, ALP, LDH) and oxidative stress (MDA, SOD, CAT) were performed.

**Results:** Rats in HFD and DSS + HFD group displayed increase in the body weight, liver weight, lipids accumulation and the levels of TNF-α, ALT, AST, ALP, MDA in serum and liver accompanied with impaired glucose tolerance, obvious hepatitis, and decreased levels of SOD and CAT in serum and liver compared to those in control group. Moreover, in the DSS + HFD group, but not in the HFD group, proliferation of fibrous tissue in the portal area and the hepatic lobules was found.

**Conclusion:** The addition of DSS on high-fat diet did not exacerbate lipid accumulation and inflammation, but induced NASH-related liver fibrosis.

## Introduction

Non-alcoholic fatty liver disease (NAFLD) is a major type of metabolic disorder, a severe public health problem, has various disease stages including simple fatty liver, non-alcoholic steatohepatitis (NASH), liver fibrosis, cirrhosis, and eventually hepatocellular carcinoma (Mazzolini et al., 2020). NASH is characterized with steatosis, lobular inflammation and hepatocellular ballooning (Kaminsky-Kolesnikov et al., 2020). Globally, the incidence and prevalence of NASH are on rise. In the developed world, NASH is the leading chronic liver disorder with an estimated prevalence of 30%. In Asia, the prevalence of NASH is up to 27% in the general population (Dennis et al., 2020). NASH attracts great attention as an important risk factor for liver fibrosis and cirrhosis. Available data show that approximate 15%–40% of NASH develops to hepatic fibrosis whereas 20% of NASH progresses to cirrhosis (Younossi, 2019). It has become the dominant cause of end-stage liver disease or of liver transplantation. Therefore, it is crucial to clarify its complex pathological process and establish its effective therapeutic strategy.

Due to the lack of an ideal experimental animal model, the pathogenesis of NASH remains yet to be elucidated. Further, development of drugs against NASH is still in a relatively slow progress. Therefore, animal models that recapitulate NASH’s clinical features continue to be needed. The available animal models of NASH include genetic models, nutritional models, and composite nutritional as well as genetic models (Solinas et al., 2014). However, the currently available animal models are limited in that they incomprehensibly or only partially reflect the etiology, pathogenesis, and mechanisms underlying the human NASH and hence restricts their application. Generally, nutritional models of NASH are induced by an unbalanced diet such as HFD, high-sucrose high-fat diet, or methionine-choline deficient (MCD) diet (Lee et al., 2019). Accumulation of lipid in hepatocytes leads to steatosis, inflammation, and fibrosis in nutritional models (Hohenester et al., 2020). MCD diet can induce hepatic damage and this can be reversed by resuming the normal diet in a short period (Itagaki et al., 2013). However, MCD diet often leads to hypoglycemia and weight loss without peripheral insulin resistance (Ozawa et al., 2018). This, shows that this animal model induced by MCD diet is more applicable to study lean NASH with the different metabolic profile as compared with that of obese NASH. Further, it has been shown that HFD can induce the metabolic profile of obese NASH with milder damage in liver as compared with the MCD diet-induced NASH model. However, it has been shown that chronic low-grade inflammation and minor fibrotic lesion in rodent liver induced by HFD are dependent on the species and strains. Previous studies have shown that NASH phenotype fails to be induced by long-term taking of HFD in Wistar rats (Van Herck et al., 2017) and more hepatic lipids accumulates in BALB/c male mice than in C57BL/6 male mice (Sanches et al., 2015) upon long-term HFD feeding. In addition, the metabolic disorders characteristic of the metabolic syndrome, such as the increased lipogenesis, stimulation of VLDL secretion, and increased plasma triglyceride levels in rats fed with high-fructose-high-fat diet are more serious than fed with HFD alone (Lin et al., 2017), which indicated that appropriate combination provides a reproducible way to induce a severe NASH phenotype. Obviously, nutritional models have some disadvantages, such as mild lesion, lengthy procedure, and higher costs.

Intestinal mucosal barrier dysfunction is common in liver diseases. Studies have shown that disruption of the intestinal epithelial barrier and gut vascular barrier contribute to the inflammatory pathway involved in NASH development. They may be early events in the development of NASH (Mouries et al., 2019). Changes in both the microbiota and gut barrier contribute to experimental NASH. Dextran sulfate sodium (DSS) is a polyanionic derivative of dextran synthesized via esterification with chlorosulfonic acid. DSS mediates direct damage to the intestinal epithelial cells, particularly in the distal colon, resulting in disruption of the intestinal barrier and cytokine production by the intestinal epithelial cells (Fan et al., 2012). Disruption of the intestinal mucosal barrier function and increased mucosal intestinal permeability causes bacterial translocation as well as endotoxemia, which then leads to liver injury through the gut-liver axis (Trivedi and Jena, 2013a; Chen et al., 2020). Liver injury can accelerate fatty acid flux to the liver, leading to increased levels of the hepatic content of total cholesterol (TC) and triglycerides (TG) (Wang et al., 2019), which eventually causes NASH. In addition, it has been evident that DSS colitis promotes tumorigenesis in a HFD-induced NASH mice model (Sato et al., 2021). In this context, combined administration of HFD and DSS induced NASH has been developed. In this study, we aim to evaluate whether this composite NASH animal model is more ideal than that induced by HFD alone.

## Materials and methods

### Animal model

Thirty male Sprague Dawley (SD) rats weighing 200 ± 50 g were purchased from the Shanghai Xipuer-Bikai Laboratory Animal Co., Ltd. (Shanghai, China). All rats were raised in the Animal Experimental Center of Shanghai University of Traditional Chinese Medicine at a temperature range between 21 and 25°C, environmental humidity of 50% ± 5% and light/dark cycle of 12 h. Approval of the experiments protocol was provided by the Animal Ethics Committee of Shanghai University of Traditional Chinese Medicine (Ethical Accreditation No. PZSHUTCM190628022). The animals were divided into three groups with 10 rats in each group: control group, HFD group and HFD combined with DSS drinking (DSS + HFD) group. Rats in control group were fed on regular chow and given normal water; Rats in HFD group were fed on HFD (D12492, Research Diet, Rodent Diet with 60% kcal from fat); Rats in DSS + HFD group were fed on HFD with 1% DSS (MP Biomedicals, OH, United States) in their drinking water. Rats received 1% (w/v) DSS dissolved in the drinking water for 7 days, followed by normal water for 10 days.

### Samples collection and processing

After 22 weeks of treatment, rats were fasted overnight before sample collection and tissue assays. Blood samples were obtained from the abdominal aorta. The blood was centrifuged and the serum was obtained for lipid profiling and inflammation measurement. A portion of the visceral fat, liver and gut tissues was fixed in 4% paraformaldehyde, and the other was fixed in formalin for routine histological examination. The remaining tissue were collected and then frozen in liquid nitrogen and stored at −80°C.

### Oral glucose tolerance test

At the last week, for oral glucose tolerance tests (OGTT), animals were fasted overnight for 12 h, and blood samples were taken from the tail vein. Fasting blood glucose was detected by touch Ultra glucometer (ONETOUCH Ultravue, China). D-glucose (Signma–Aldrich) was given using an oral gavage at a dose of 2 g/kg (25% w/v glucose solution). Blood glucose was measured in the tail vein blood at 15, 30, 60, 90, and 120 min. Total area under the curve (AUC) were lastly used to evaluate the glucose tolerance of the rats during the OGTT.

### Histological examination

Hematoxylin and eosin (HE) staining was used to observe the general histological pathology. Masson staining was used to visualize fibrosis in liver. For HE staining and Masson, tissue blocks were prepared for sectioning at 4 μm thickness by slidge microtome. Tissues were paraffin-embedded, dewaxed, rehydrated, and stained with HE solution (G1005, Servicebio) and Masson (PH1427, Servicebio) according to the respective manufacturers’ instructions provided.

Oil red O staining was used to visualize hepatic lipid accumulation. A stock of oil red O solution (Sigma, St. Louis, United States) was prepared through combination of 150 mg of oil Red O powder with 50 ml of isopropanol. The Oil Red O working solution was made fresh on the day of staining by diluting the stock solution with ddH_2_O in a ratio of 3:2, and the working solution was filtered before use. The wells were washed with sterile PBS and stained with Oil Red O working solution for 30 min at room temperature. The Oil Red O solution was then removed, and the wells were washed thrice with PBS to remove the excess stain. Finally, the nucleus was stained with hematoxylin solution for 30 s and then rinsed with tap water.

Liver sections were sealed with a neutral resin, and observed and photographed under a microscope. The liver slides were then assessed for steatosis, inflammation and fibrosis, using the NAFLD activity score (NAS). The score was calculated based on the individual scores of steatosis (0–3 points), lobular inflammation (0–3 points) and hepatocyte ballooning (0–2 points) in a blinded manner. By definition, a NAS  score <3 was not regarded to be NASH condition, whereas a NAS score ≥5 score represents NASH (Kleiner et al., 2005).

### Biochemical parameters

Serum glucose, lipid profiles, and liver biochemical analysis were determined by using a Beckman Coulter Immage800 automatic biochemical analyzer (Beckman Coulter, Inc., United States). Blood sugar level was assessed by circulating glucose (GLU), and lipids by non-esterified fatty acids (NEFA), high-density lipoprotein (HDL), low-density lipoprotein (LDL), triglycerides (TG) and total cholesterol (TC). Liver function was evaluated by measuring the liver function indices that included aspartate aminotransferase (AST), alanine aminotransferase (ALT), alkaline phosphatase (ALP) and lactate dehydrogenase (LDH).

### Enzymatic colorimetric assays

Precooled absolute ethanol lysate was added at mass/volume ratio of 1:9 to a 100 mg of liver tissue for homogenization. The tissue homogenate of 10% liver tissue was prepared through its full grinding in ice water bath with high-speed disperser (Servicebio, China). Supernatant was taken for later analysis. Liver TG and TC were analyzed using enzyme kits (A110-1; A111-1-1; Nanjing Jiancheng Institute of Biological Engineering, China) according to the manufacturer’s instructions. Liver TG and TC were analyzed with enzyme kits (Nanjing Jiancheng Institute of Biological Engineering, China) according to the manufacturer’s instructions.

Serum TNF-α was measured using enzyme-linked immunosorbent assay (ELISA) kit (ml002953; Shanghai mlbio, China) according to the manufacturer’s instructions. Tissue oxidative stress was assessed with kits for MDA, SOD and CAT assays (ml077384; ml059387; ml037079; Shanghai mlbio, China). Livers were homogenized with saline to make a 10% homogenate. The serum or liver homogenate were incubated with biotinylated antibodies and horseradish peroxidase at 26°C for 60 min. After washing, the substrate reagent was mixed with the chromogen and incubated for 15 min. The OD value at 450 nm was measured with a microplate reader.

### Statistical analysis

The SPSS software (version.24.0) and GraphPad Prism 8.2.1 were used to carry out the statistical analysis of the data obtained in the present study. Data are expressed as mean ± SD, with normality and variance homogeneity tested. The Student’s t-test was used to analyze individual differences. If data were not under normal distribution, they were analyzed by the Wilcoxon rank sum test. *p* < 0.05 was considered statistically significant.

## Results

### Effects of HFD combined with DSS on OGTT, body weight, liver weight, and liver index

**FIGURE 1:**
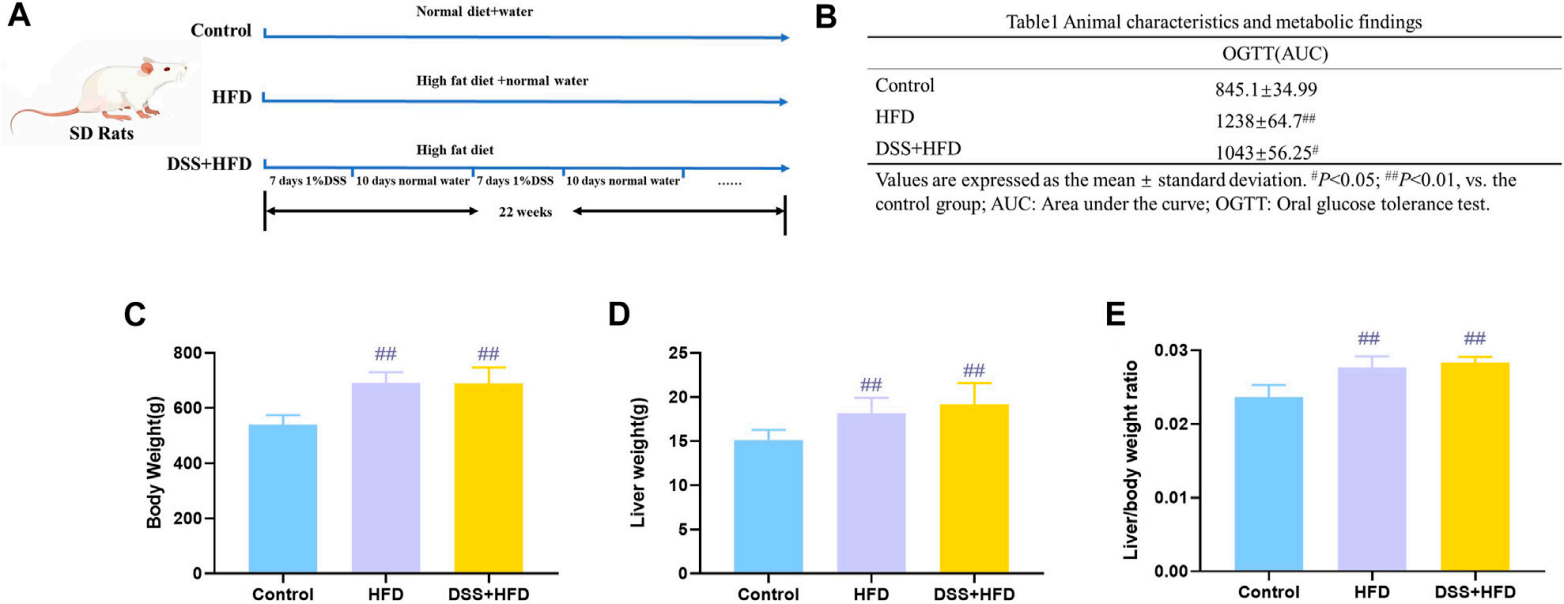
Metabolism of rats during modeling. **(A)** Methodology flow chart. **(B)** Body weight. **(C)** Liver weight. **(D)** Liver/body weight ratio. Values are means ± SD (*n* = 4–6). Control: standard chow; HFD: high fat diet; DSS + HFD: high fat diet +1% DSS in drinking water. ^#^ *p* < 0.05, ^##^ *p* < 0.01, vs. the control group.

The schedule with different feeding methods is shown in Figure 1A. Generally, the rats in each group had free access to water and food during the entire modeling period. AUC for the OGTT in the HFD, DSS + HFD and control groups were 1,238 ± 64.7, 1,043 ± 56.25 and 845.1 ± 34.99, respectively. The OGTT results showed the impairment of glucose tolerance in rats fed high-fat diet. Compared with the DSS + HFD group, the HFD group showed a more severely impaired glucose disposal at 22 weeks (Table 1). The initial body weights of all groups were similar. The HFD group and the DSS + HFD group gained more weight over the 22 weeks compared with the control group. And the rats fed HFD had a marked increase in final body weight, liver weight and in the liver index (liver wet weight/body weight × 100%) compared with the control rats (^##^ *p* <0.01, vs. the control group, respectively) (Figures 1B–D). Of note, there was no difference in body weight, liver weight or liver to body weight ratio between HFD group and HFD + DSS group (*p* > 0.05). (Figures 1B–D).

**Table 1:** Animal characteristics and metabolic findings.

	OGTT(AUC)
Control	845.1±34.99
HFD	1238±64.7^##^
DSS+HFD	1043±56.25^#^

Values are expressed as the mean ± standard deviation ^#^ *p* < 0.05; ^##^ *p* < 0.01, vs. the control group AUC; Area under the curve; OGTT; Oral glucose tolerance test.

### Lipid concentration in serum and liver

**FIGURE 2:**
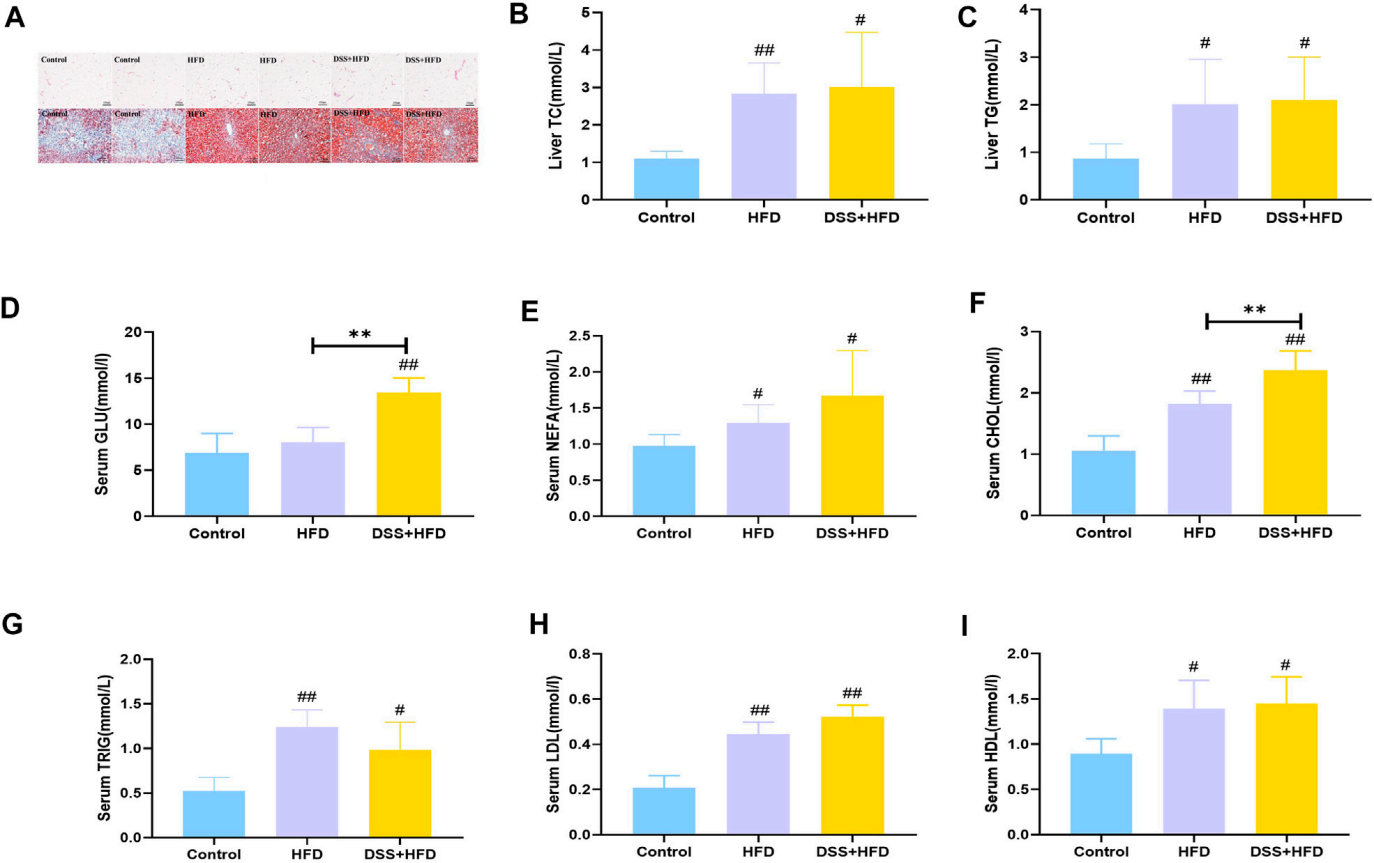
Lipid metabolism indices in each group. **(A)** HE staining of visceral adipose. Oil-Red O staining of liver. Pictures were taken using a microscopy with ×10 object lenses. **(B)** Hepatic TG contents. **(C)** Hepatic T-CHO contents. **(D)** GLU. **(E)** NEFA. **(F)** CHO. **(G)** TRIG. **(H)** LDL. **(I)** HDL. Values are the means ± SD (*n* = 5). Control: standard chow; HFD: high fat diet; DSS + HFD: high fat diet + 1% DSS in drinking water. ^#^ *p* < 0.05, ^##^ *p* < 0.01, vs. the control group. **p < 0.01, vs. the HFD group.

The visceral adipocytes in the HFD and the DSS + HFD groups were significantly larger than the control group (Figure 2A). What’s more, adipocytes of rats in the HFD and the DSS + HFD groups exhibited uneven sizes and abnormal distributions. We stained the liver tissues with Oil-red O staining to visualize lipid droplets. There was no obvious lipid droplet staining with the control group, while lipid droplets varied in size and were scattered around the hepatic lobules of the HFD group. In the DSS + HFD group, the sizes of the lipid droplets were significantly reduced compared to the HFD group. Also, variable degrees of hepatocytes cytoplasmic vacuolation were observed in the HFD and the DSS + HFD groups where fat vacuoles pushed the nuclei to the periphery. Liver levels of TC, TG and serum levels of GLU, NEFA, CHOL, TRIG, LDL and HDL are shown in Figures 2B–I. Compared with the control group, serum CHOL and TRIG, as well as hepatic TG and TC were significantly increased in the DSS + HFD group (^#^ *p* < 0.05 and ^##^ *p* < 0.01, vs. the control group, respectively). The concentration of serum CHOL was increased in the DSS + HFD group, when compared to the HFD group (**p < 0.01, vs. the HFD group). There was no significant difference between the HFD and the DSS + HFD groups concerning serum TRIG, hepatic TG and TC. Serum GLU levels was significantly increased in the DSS + HFD group compared with the control group (^##^ *p* < 0.01, vs. the control group). However, GLU levels were not different between the control and the DSS + HFD groups. Also, there were increased levels of NEFA, LDL and HDL in the HFD and the DSS + HFD groups (^#^ *p* < 0.05 and ^##^ *p* < 0.01, vs. the control group, respectively). And no significant difference was found in the levels of NEFA, LDL and HDL between the HFD and the DSS + HFD groups.

### HFD combined with DSS caused inflammation

**FIGURE 3:**
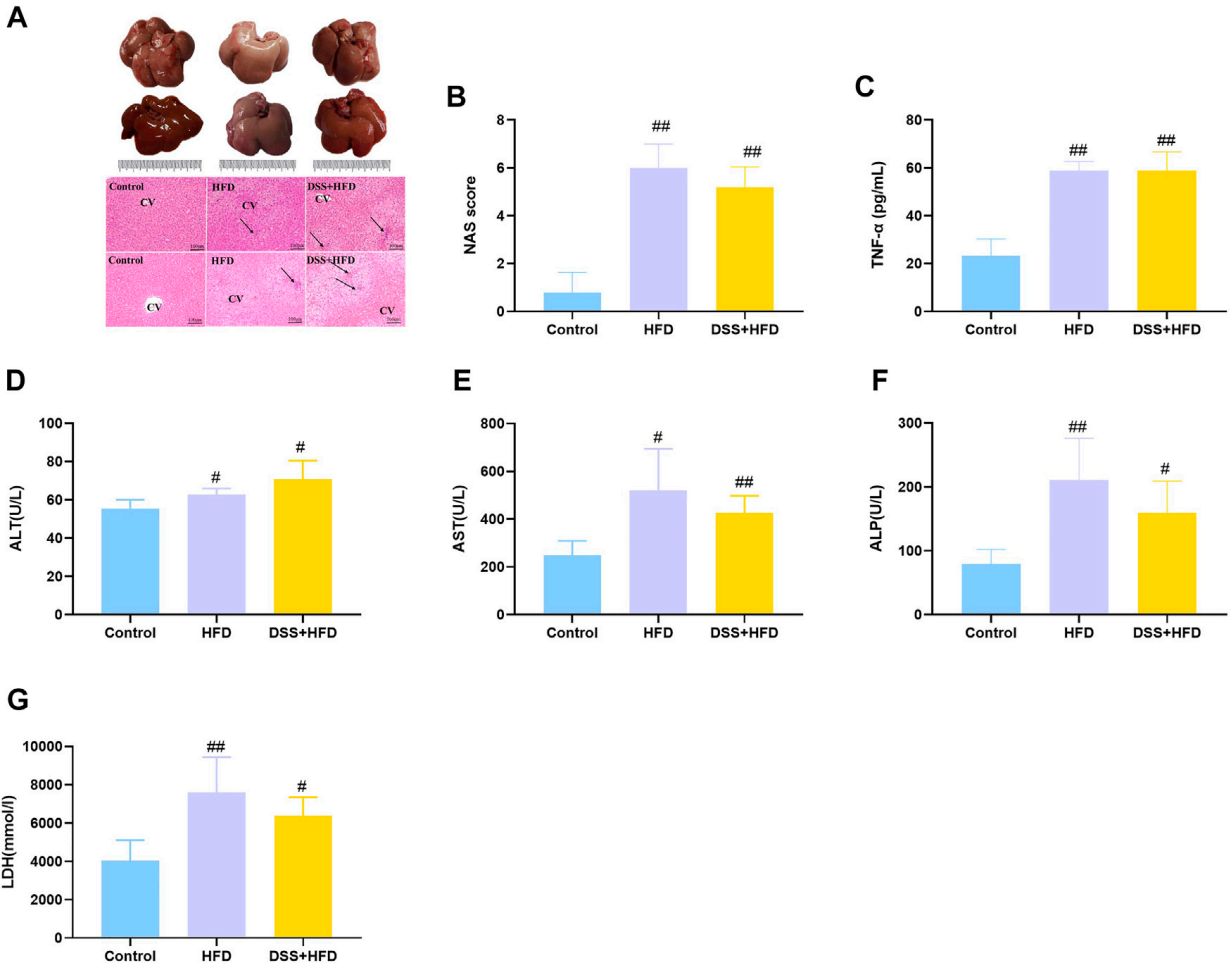
Evaluation of hepatic steatosis. **(A)** Macroscopic liver appearance and liver histology as shown by HE staining of liver. Pictures were taken under a microscopy with object lenses of ×10. Black arrows point at inflammatory cells. CV, central vein **(B)** NAFLD Activity Score (NAS) analysis. **(C)** TNF-α. **(D)** ALT. **(E)** AST. **(F)** ALP. **(G)** LDH. Values are the means ± SD (*n* = 5). Control: standard chow; HFD: high fat diet; DSS + HFD: high fat diet +1% DSS in drinking water. ^#^ *p* < 0.05, ^##^ *p* < 0.01, vs. the control group. **p < 0.01, vs. the HFD group.

Histopathological changes in the liver tissue of various groups are summarized in Figure 3A. The control livers were reddish brown while those from the DSS + HFD group were dark red. And the HFD-only intervention markedly lightened the color of the liver, which were bigger and had a yellowish-white color. Meanwhile, the whole livers in the control group were characterized by sharp edges. HE staining showed that, the hepatocytes were uniform in size and normal in nuclear morphology in the control group. The hepatic lobule and liver rope both had clear structures and regular arrangement. Hepatocytes were loosely arranged except for the rats on the normal diet. Hepatocyte size was greater in the HFD and the DSS + HFD groups than in the control group. To be specific, in the HFD group, hepatocytes showed extensive fatty degeneration and obvious swelling. In the DSS + HFD group, the hepatocytes showed irregular morphology, loss of cell boundary, cell swelling, and widespread signs of inflammatory infiltration. Together with our previous Oil Red O staining results, overall NAS score was calculated based on ballooning, inflammation, and steatosis scores. Definite steatohepatitis was diagnosed in the rats with NAS ≥5 in current study. The HFD and the DSS + HFD groups had over five points of NAS (Figure 3B). TNF-α, ALT, AST, ALP and LDH levels are indicators of liver damage (Figures 3C–G). Rats in the HFD and the DSS + HFD groups showed significantly higher levels of TNF-α, ALT, AST, ALP and LDH (^#^ *p* < 0.05 and ^##^ *p* < 0.01, vs. the control group, respectively), but no significant difference was observed between the two groups (*p* > 0.05).

### HFD combined with DSS induced oxidative stress and liver fibrosis

**FIGURE 4:**
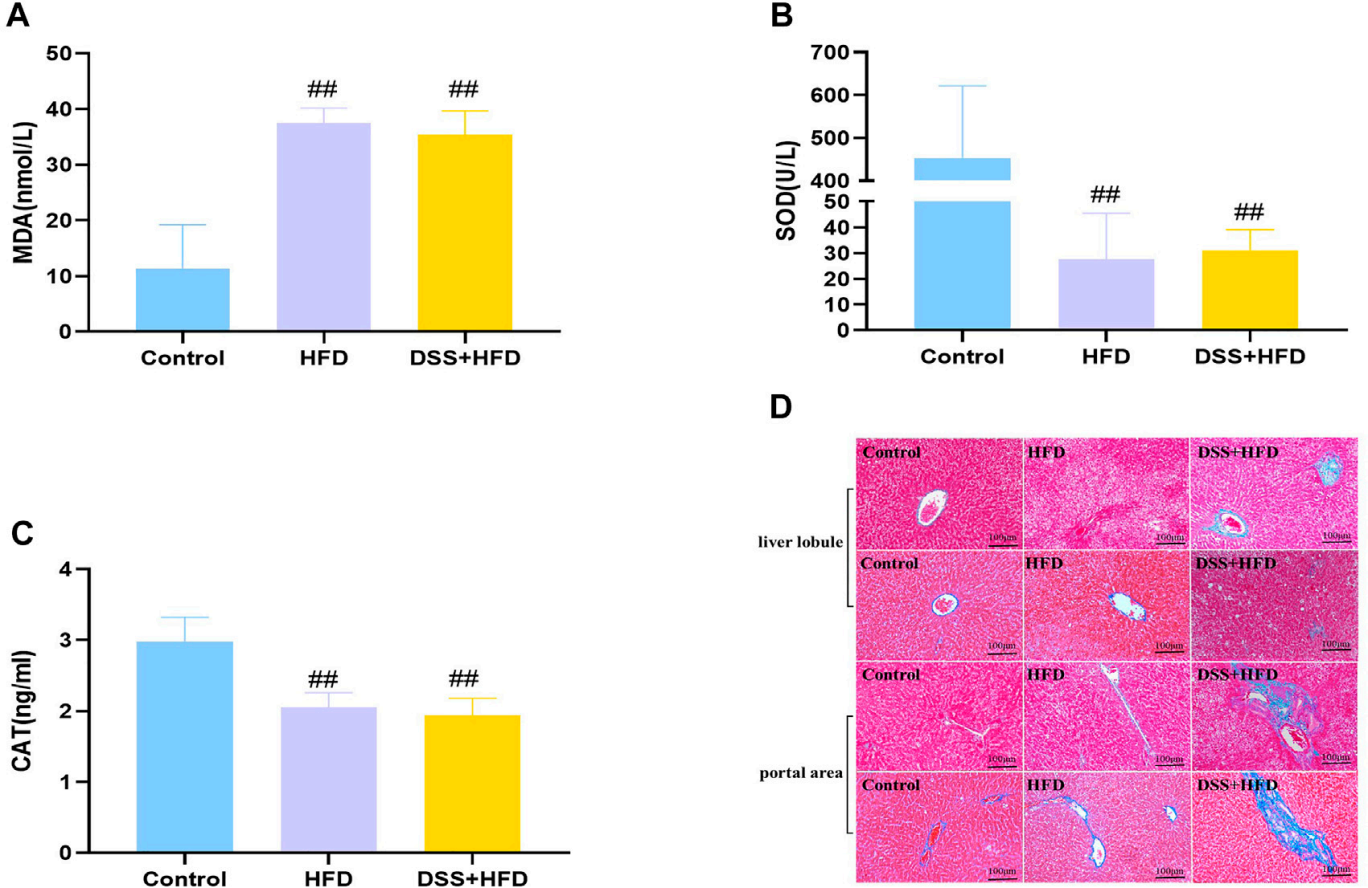
Effect of HFD and DSS interaction on levels of oxidative stress and fibrosis. **(A)** MDA contents. **(B)** SOD activity. **(C)** CAT activity. **(D)** Liver sections in the three groups were stained with Masson staining. Regions of blue staining representing collagen fiber. Pictures were taken under a microscopy with object lenses of ×10. Values are the means ± SD (*N* = 5). Control: standard chow; HFD: high fat diet; DSS + HFD: high fat diet +1% DSS in drinking water. ^#^ *p* < 0.05, ^##^ *p* < 0.01, vs. the control group.

Hepatic oxidative stress is a dominant feature of NASH. MDA was markedly higher in the HFD and the DSS + HFD groups (^##^ *p* < 0.01 vs. the control group). SOD activity and CAT content were significantly negatively correlated with MDA content. Lower levels of SOD and CAT were observed in the HFD and the DSS + HFD groups ^(##^ *p* < 0.01, vs. the control group) (Figures 4A–C). No difference was observed between the HFD and the DSS + HFD groups (*p* > 0.05).

Reactive oxygen species (ROS), indicators for oxidative stress, can be generated in large quantities, which then drive the synthesis and deposition of collagen I (Hosui et al., 2009; Piegari et al., 2020). Masson staining demonstrated that liver tissues from control group showed little collagen deposition, whereas those from the HFD group presented dense fibrous septa and increased deposition of collagen fibers. By contrast, the livers of the DSS + HFD group exhibited markedly widening fibrotic portal areas, with irregular borders forming a spiked appearance and portal-bridging fibrosis (Figure 4D).

## Discussion

To date, the successful establishment of the NASH model mimicking human disease development process, is still abscent. In the past years, studies have shown that when fructose is ingested with fat, more severe hepatic steatosis and inflammation were induced compared to HFD alone (Zaki et al., 2019). What’s more, Gäbele et al. (2011) found that DSS induced colitis enhances hepatic inflammation and fibrosis in experimental NASH. Thus, we hypothesized that the combination of high-fat diet and DSS may synergistically exacerbates NASH symptoms and shorten the time required for modeling. Surprisingly, our findings show that the DSS + HFD group did not exhibit more serious steatosis or inflammation than the HFD group. Interestingly, we observed that DSS induced hepatic fibrosis in the DSS + HFD group. Thus, this combination of HFD and DSS can mimic natural course of liver fibrosis development in NASH as seen in humans.

We initially expected a mutual promotion and vicious circle between the intestinal inflammation and liver damage in the development of NASH. Elsewhere, Kwon et al. (2021) stated that DSS-induced colitis causes an imbalance in cholesterol homeostasis and this was confirmed by the significant increases in the liver weight, hepatocytes fat deposition, as well as TG content. It has been found that the DSS-induced barrier dysfunction may cause a disturbance of inter-organ metabolic homeostasis and hence causing an impaired lipid metabolism in liver and adipose tissues (Liu et al., 2018). DSS-induced chronic colitis model with inflammation and oxidative stress causes DNA damage in the colon, aggravate fibrosis, and contribute to the development of NASH (Trivedi and Jena, 2013b). Colitis causes gut dysbiosis, increase bacterial translocation to the liver leading to hepatic damage in mice with DSS, and is associated with an increase in the level of hepatic LPS (Cheng et al., 2018; Svegliati-Baroni et al., 2020). The inflammatory factors secreted by liver can enter intestine, and aggravate the damage of intestinal lining and disrupt microbiota through the bile secretion into the intestinal lumen (Yang et al., 2020). Several shreds of evidence that liver injury diseases are causally associated with impaired intestinal permeability. In chronic colitis by DSS, goblet cells are frequently destroyed or functionally impaired and the mucus barrier is hence significantly disturbed (Hoffmann et al., 2017). According to a study conducted by Tang et al. (2019), it is evident that the defects in Paneth cells can cause an increase in bacterial penetration into the intestinal barrier after interaction with DSS. The disrupted intestinal barrier causes translocation of intestinal LPS into circulation. In addition, levels of serum LPS are elevated and associated with gut dysbiosis in patients with NASH (Liang et al., 2014; Hegazy et al., 2020). Further, LPS penetrates liver via portal circulation, so can stimulate hepatocytes and contribute to pathological progression of NASH (Yuan et al., 2019). It is evident that diet of an animal affects their intestinal microbiota composition (Jiang et al., 2016). Additionally, HFD feeding with DSS induced colitis model also contributes to the increased susceptibility to intestinal inflammation. Consumption of HFD compromises the gut barrier, facilitating the onset of obesity and associated metabolic disorders (Atarashi et al., 2018). Altogether, the above insights lend support that HFD and DSS together produced a greater effect than HFD alone.

At first glance, the outcomes in the DSS + HFD group were not anticipated. Although DSS-induced inflammation was localized to the large intestine, the function of the ileum may have been compromised due to high-fat diet. DSS could cause terminal ileum epithelial cell damage and reduced levels of the terminal ileum bile acids (BAs) and bile acid-activated nuclear receptors. BA signaling is mainly mediated by the nuclear farnesoid X receptor (FXR) and the membrane-bound Takeda G protein-coupled receptor 5 (TGR5) (Wei et al., 2020). One route for lipid disposal in the liver is through cholesterol catabolism, bile acid synthesis, and biliary cholesterol secretion (Rao et al., 2016). We found increased levels of TC, TG, LDL and HDL in the DSS + HFD group, which implied increased levels of the bile acid synthesis. Increased bile acid synthesis and transportation leads to activation of FXR. Nuclear receptor FXR is involved in a negative feedback loop. Recently, it was shown that FXR deficiency in mice prevents genetic and diet-induced obesity, insulin resistance, and NAFLD (Prawitt et al., 2011; Gonzalez et al., 2016), while long-term administration of the synthetic FXR agonist GW4064 leads to lipid accumulation in liver in mice fed high-fat diet (Watanabe et al., 2011). The gut microbiota promotes diet-induced obesity and associated phenotypes through FXR (Parséus et al., 2017). The ileum farnesoid X receptor-fibroblast growth factor 15 (FXR-FGF15) axis was downregulated after co-treating with azoxymethane (AOM) and DSS (Liu et al., 2021). DSS can reduce bile acid concentrations in the intestine by activation of FXR. Rats with decreased FXR expression in the intestine were resistant to HFD-induced NASH. Should this be the case, it could partly explain the phenomenon that the combination of HFD and DSS could not generate a synergistic effect.

Indeed, combining the high-fat diet fed NAFLD model and the chronic DSS colitis model did not yield superposed effects. But it is undeniable that we still established the NASH model and recapitulates the phenotype of NASH in humans. We get a less severe phenotype in terms of lipid accumulation than the classical high fat induced NASH model, but it is significantly more severe in terms of fibrosis. It would be interesting to utilize different NASH models and probe into some universal phenotype. Combined high-fat diet and DSS model may fulfill the unmet need for studying the progression from NASH to fibrosis.

## Data Availability

The original contributions presented in the study are included in the article/Supplementary Material, further inquiries can be directed to the corresponding authors.
